# Adolescents with current major depressive disorder show dissimilar patterns of age-related differences in ACC and thalamus

**DOI:** 10.1016/j.nicl.2014.12.019

**Published:** 2015-01-07

**Authors:** Cindy C. Hagan, Julia M.E. Graham, Roger Tait, Barry Widmer, Adrienne O. van Nieuwenhuizen, Cinly Ooi, Kirstie J. Whitaker, Tiago Simas, Edward T. Bullmore, Belinda R. Lennox, Barbara J. Sahakian, Ian M. Goodyer, John Suckling

**Affiliations:** a Department of Psychiatry, University of Cambridge, Cambridge, UK; b Department of Psychology, Columbia University, New York, NY, USA; c MRC/Wellcome Trust Behavioural and Clinical Neuroscience Institute, University of Cambridge, Cambridge, UK; d Department of Psychiatry, University of Oxford, Medical Sciences Division, Oxford, UK

**Keywords:** Depression, Adolescence, Thalamus, Cingulate, Voxel-based morphometry, MRI

## Abstract

**Objective:**

There is little understanding of the neural system abnormalities subserving adolescent major depressive disorder (MDD). In a cross-sectional study we compare currently unipolar depressed with healthy adolescents to determine if group differences in grey matter volume (GMV) were influenced by age and illness severity.

**Method:**

Structural neuroimaging was performed on 109 adolescents with current MDD and 36 healthy controls, matched for age, gender, and handedness. GMV differences were examined within the anterior cingulate cortex (ACC) and across the whole-brain. The effects of age and self-reported depressive symptoms were also examined in regions showing significant main or interaction effects.

**Results:**

Whole-brain voxel based morphometry revealed no significant group differences. At the whole-brain level, both groups showed a main effect of age on GMV, although this effect was more pronounced in controls. Significant group-by-age interactions were noted: A significant regional group-by-age interaction was observed in the ACC. GMV in the ACC showed patterns of age-related differences that were dissimilar between adolescents with MDD and healthy controls. GMV in the thalamus showed an opposite pattern of age-related differences in adolescent patients compared to healthy controls. In patients, GMV in the thalamus, but not the ACC, was inversely related with self-reported depressive symptoms.

**Conclusions:**

The depressed adolescent brain shows dissimilar age-related and symptom-sensitive patterns of GMV differences compared with controls. The thalamus and ACC may comprise neural markers for detecting these effects in youth. Further investigations therefore need to take both age and level of current symptoms into account when disaggregating antecedent neural vulnerabilities for MDD from the effects of MDD on the developing brain.

## Highlights

• Adolescents with MDD show no group differences in GMV compared with controls.
• MDD show dissimilar patterns of age-related GMV differences in ACC than controls.
• Opposing case–control patterns of age-related GMV differences are shown in thalamus.
• GMV differences in thalamus were evident following whole brain examination.
• In MDD, thalamic, but not ACC, GMV inversely correlates with depressive symptoms.

## 1 Introduction

The emergence of major depressive disorder (MDD) in adolescence is associated with poorer lifelong health outcomes for the individual which may, in part, be related to suboptimal brain maturational processes (Paus et al., 2008). This observation is coupled with evidence showing that despite remittance of depression, traces of the illness may remain in the brain (sometimes termed neural residue) rendering an individual more vulnerable to subsequent episodes (Kendler et al., 2000; Post, 1992). Early detection and treatment, a therapeutic goal for clinicians, may serve to minimise these neural traces and therefore reduce the neural risks accruing from depression in youth.

One strategy for further understanding the pathophysiology of MDD is to examine brain structural differences between affected and unaffected young persons prior to treatment. Endocrine, psychological, neuroimaging and post-mortem examinations of MDD have implicated the hypothalamic–pituitary–adrenal axis, cortico-striatal-pallidal-thalamic, amygdalo-striato-pallido-thalamic, default mode, reward system, cortico-basal ganglia circuit, orbital prefrontal and medial prefrontal (which incorporates the fronto-limbic system) networks (Wilkinson and Goodyer, 2011; Graham et al., 2013; Roiser et al., 2012; Gotlib et al., 2010; Marchand and Yurgelun-Todd, 2010; Price and Drevets, 2012). While several components of these networks, including the pre- and subgenual anterior cingulate cortex (pgACC; sgACC), have shown reduced gray matter volume (GMV) in adults with depression (reviewed in Price and Drevets, 2012), a recent meta-analysis revealed that GMV reductions in the pregenual ACC are the most consistent finding across whole-brain voxel-based morphometry (VBM) studies of MDD in adults (Bora et al., 2012). Notably, the meta-analysis failed to find amygdala or hippocampal regional differences in GMV in individuals with MDD (Bora et al., 2012), in line with a recent large study of adult MDD that also failed to find group differences in limbic regions (Grieve et al., 2013). We therefore restricted our focus to the ACC region only.

Using a region of interest (ROI) approach and manual tracing techniques, a recent study of adolescents with current depression revealed significantly smaller GMV in the ACC in comparison to healthy controls (MacMaster et al., 2014). Furthermore, at trend-level significance, GMV reductions in the ACC differentiated MDD from bipolar disorder (MacMaster et al., 2014). Researchers noted that male adolescents mostly drove the effect in the ACC, despite no observable sex-differences within the MDD group alone (MacMaster et al., 2014). Interestingly, a recent prospective longitudinal study of a community sample of adolescents prior to and following their first depressive episode failed to observe a significant main effect of group or group-by-time interactions in the ACC (Whittle et al., 2014) The different results observed across the two studies may have been attributable to the relatively small sample size, given the heterogeneity of MDD. Thus, it is unclear whether similar differences in brain structure can be extended to a much larger sample of adolescents who are currently depressed.

While not much is known about the neurobiological pathophysiology of depression in adolescence, a considerable reorganisation of grey and white matter is known to occur in the healthy adolescent brain (Lenroot and Giedd, 2006). Gender may play a role in adolescent brain development as total brain volume, which encompasses grey and white matter and cerebrospinal fluid, peaks earlier for girls than for boys, with boys, on average, possessing larger brains than girls irrespective of height and weight differences (Lenroot and Giedd, 2006). With respect to GMV, different curvilinear patterns of development (often described as an inverted U) may characterise the growth of different brain regions (Grieve et al., 2013), with phylogenetically older brain regions maturing at relatively younger ages than regions more recently evolved (Lenroot and Giedd, 2006; Gogtay et al., 2004). Thus, when identifying structural changes in the depressed adolescent brain, studies should account for gender and age-related changes in structural brain development in order to minimise false positives.

To better understand the neural changes associated with MDD in adolescence, we examined cross-sectional differences in brain structure associated in a large sample of adolescents compared with healthy age- and gender-matched controls. Acknowledging that first episodes occur prior to age 18 years in an estimated 25% of adult individuals with mood disorders and that age (and associated neurobiological changes) plays a key role in the aetiology of mental illness (Jones, 2013), and depression in particular (Whittle et al., 2014), we examined differences in GMV between groups of adolescents with current depression and and healthy controls taking the putative effects of age on GMV into account (Paus et al., 2008). Given that the most common finding in adults with MDD is reduced GMV in pregenual ACC (Bora et al., 2012), a region important to both the onset and course of depressive illness (Chen et al., 2007), we predicted that adolescents with MDD would show reduced GMV in this region relative to healthy adolescents irrespective of age-dependent change. We conjecture that the ACC is a sensitive marker of the neural impact of illness irrespective of the age of the patient.

In adolescence, reduced GMV implies relative maturity in many regions of the adolescent brain apart from the temporal lobes (Lenroot and Giedd, 2006). We acknowledge, however, that adverse experiences in early childhood are more often associated with onsets of depression in early as opposed to late adolescence (Andersen and Teicher, 2008), and that genetic factors are more often associated with onsets of depression in late as opposed to early adolescence (Peterson et al., 2014), we speculate that an alternate hypothesis can be tested whereby the effect of MDD on GMV may be inversely related to age. This hypothesis would hold that younger aged depressed individuals are more prone to accelerated maturation of this region than their older ill counterparts. If true, then we would predict a significant group × age interaction with younger MDD cases showing the strongest effects of illness on the ACC.

We have previously shown a non-depressive comorbidity prevalence of approximately 50% for MDD patients attending UK-NHS Clinics (Goodyer et al., 2008) with the most common comorbid diagnoses being anxiety disorders. Furthermore a general factor of distress appears to underlie the reporting of both depressive and anxiety symptoms in this age range (Brodbeck et al., 2014). Consequently, we included self-reported measures of depressive symptoms and also trait anxiety. As researchers suggest that the neural traces of depression may develop in proportion to the severity of each depressive episode (Wichers et al., 2010; Robinson and Sahakian, 2008), we also tested the hypothesis that higher self-reported symptom levels would be associated with greater reductions in GMV in the ACC Independent of age.

## 2 Materials and methods

### 2.1 Subject characteristics

This study, Magnetic Resonance — IMPACT (MR-IMPACT), was an adjunct to the Improving Mood with Psychoanalytic and Cognitive Therapies (IMPACT) clinical trial (Goodyer et al., 2011) from which subjects with moderate to severe depression were recruited. Full details of the MR-IMPACT protocol are given in Hagan et al. (2013) but, briefly, all patient subjects met DSM-IV criteria for current MDD (presence of 1 core symptom and 4 additional depressive symptoms in the 2 weeks prior to interview), as determined by combining information from child and parent interviews using the Kiddie Schedule for Affective Disorders and Schizophrenia — Present and Lifetime version (K-SADS-PL; Kaufman et al., 1997). Self-report depression scores using the Mood and Feelings Questionnaire (MFQ), obtained at the time of research assessment for the trial reveal a mean of 45.6 (S.D. 10.5) out of a maximum possible score of 66; the index of caseness for depressions is taken as a score of >26 (Angold et al., 1987).

General exclusion criteria for IMPACT and MR-IMPACT included the presence of one or more of the following: alcohol or drug abuse or dependence; pervasive developmental disorder or generalised learning problems resulting in an inability to complete study questionnaires; current and necessary use of medication with possible adverse interactions with SSRIs; presence of MRI contraindication or brain abnormality (either known beforehand or identified by a neuroradiologist via axial proton density T2 or axial flair images); or intolerance to the MRI environment. All participants and their families provided written informed assent and consent, with procedures carried out in accordance with the Declaration of Helsinki. (World Medical Association, World Medical Association, 2008) Ethical approval for the study was granted by the National Research Ethics Service Committee East of England – Cambridge Central.

Table 1 presents the demographic data of the MR-IMPACT cohort. All demographic data were analysed using version 19 of the Statistical Packages for the Social Sciences (SPSS; IBM Corporation, New York). Independent sample *t*-tests were performed to explore group differences in all demographic variables, with the exception of categorical data on which chi-square tests were performed. Where Mauchly's test indicated a violation of sphericity, Greenhouse–Geisser corrections were applied. All tests were performed with levels of significance established at *p* < 0.05, two-tailed.

Considering the potential effects of clinical heterogeneity on any results, we included a substantial number of adolescents with current DSM IV MDD (unipolar) episodes in an effort to increase power and thus our ability to detect brain structural differences that we could interpret as associated with this psychiatric syndrome. In total, 128 patients with unipolar major depression aged 11–17 years were recruited to MR-IMPACT from NHS clinics in the East Anglian (*n* = 110) and North London regions (*n* = 18) of the UK. Nineteen were excluded for one of three reasons: 2 for brain abnormalities; 11 for brace or retaining wire MRI artefacts; 6 for scans occurring after psychological treatment had commenced. Thus, 109 adolescents with depression were included in the overall analysis (81 female, 28 male, mean (M) age ± standard deviation (SD) = 15.56 ± 1.27, age range = 11.83–17.96). On average females were older (M = 15.68, SD = 1.14) than males (M = 15.22, SD = 1.57), however, this difference was not significant *t*(107) = −1.65, *p* = 0.10 and represented a small sized effect *r* = 0.16.

Approximately one third of these patients were taking antidepressant medication at the time of scanning and all were randomised to one of three possible types of active psychological treatment (specialist clinical care, cognitive behaviour therapy, or short-term psychoanalytic therapy). Antidepressant medication was restricted to selective serotonin reuptake inhibitors (SSRIs), with fluoxetine the most common prescribed medication (Table 1). Of the 37 medicated subjects with depression, 24 were female and 13 were male (M ± SD = 15.71 ± 1.35). Again, females were older on average (M = 15.92, SD = 1.18) than were males (M = 15.33, SD = 1.61). This difference was not significant *t*(35) = −1.28, *p* = 0.21 and represented a small effect size, *r* = 0.21.

Forty healthy young persons, matched for age, gender, and handedness, were recruited from local Cambridgeshire schools by study advertisement to the students and their parents. Four were excluded due to brain abnormality, pre-existing medical condition, or brace/retaining wire artefact, leaving 36 control subjects (26 female, 10 male, M ± SD = 15.65 ± 1.45, age range = 12.14–17.73); see Table 1 for details. Healthy control females were, on average, older (M = 15.81, SD = 1.52) than males (M = 15.25, SD = 1.22), however, this difference was not significant *t*(34) = −1.03, *p* = 0.31 and represented a small effect size, *r* = 0.17. Control participants met the same inclusion criteria applied to the depressed group, with the exception of screening for lifetime history of mood disorder or substance dependence in first-degree relatives or a personal history of psychopathology requiring clinical treatment. Control participants were excluded if they had either a lifetime history of psychopathology requiring clinical treatment, or a history in the immediate family of mood disorder or substance dependence. Control participants also were screened with the self-report Moods and Feelings Questionnaire — Short version (SMFQ) and were required to obtain a score ≤5 which is less than the clinical cutoff of ≥8 (Angold et al., 1995).

For a simple two-group comparison of means (i.e., a *t*-test), the power to detect a fixed effect size is a function of the ratio of participants in each group. Given the sample sizes for each group, we estimate our power for detecting true significant effects at 0.70.

### 2.2 Structural MRI (sMRI) data acquisition

MR images of all subjects were acquired by a 3.0 Tesla Magnetom Trio Tim scanner (Siemens, Surrey, England) fitted with a quadrature birdcage head coil at the Wolfson Brain Imaging Centre, University of Cambridge, UK. A T1-weighted image was acquired in the sagittal plane using a three-dimensional magnetically prepared rapid acquisition gradient echo sequence (3D-MPRAGE; 176 slices of 1.00 mm thickness, echo time = 2.98 ms, repetition time = 2.30 s, inversion time = 900 ms, flip angle = 9°, field of view = 240 × 256 mm^2^, voxel size = 1.0 × 1.0 × 1.0 mm^3^, series = interleaved).

Brain abnormalities were screened with a high-resolution dual echo — proton density and T2-weighted sequence by a senior neuroradiologist.

### 2.3 Pre-processing and statistical analysis of sMRI data

Whole brain VBM was first used to identify pre-treatment anatomical differences between patients and controls. Voxelwise estimates of GMV from each participant's T1-weighted images were calculated using an optimised VBM protocol (Good et al., 2001) using version 5 of FSLVBM (Smith et al., 2004); http://fsl.fmrib.ox.ac.uk/fsl/fslwiki/FSLVBM) from the Functional Magnetic Resonance Imaging of the Brian software library (FSL; Douaud et al., 2007).

Structural images were first skull-stripped using the brain extraction tool (Smith, 2002) and then segmented into grey and white matter tissue types using FAST (Zhang et al., 2001). Grey matter partial volume images from a subset of 72 subjects (36 subjects from each group, matched for age, gender and handedness) were used to generate a study specific template against which all remaining native grey matter partial volume images were subsequently non-linearly re-registered.

Grey matter partial volume images from the subset of subjects were first aligned to Montreal Neurological Institute (MNI) standard space using the affine linear registration tool FLIRT (Jenkinson and Smith, 2001; Jenkinson et al., 2002) followed by non-linear registration using FNIRT (Andersson et al., 2007). The resulting images were processed to create a left–right symmetric, study-specific grey matter template. All native space grey and white matter images were then non-linearly registered to the respective study-specific grey matter and white matter template and modulated to control for local expansion or contraction. The modulated GMV images were then smoothed with an isotropic Gaussian kernel with a sigma of 3 mm.

#### 2.3.1 Statistical analysis of sMRI data

##### 2.3.1.1 Whole brain voxel-based analysis

Gender was modelled as a nuisance covariate. We opted against modelling either total intracranial volume (TIV) or total GMV as a nuisance covariate as it is strongly collinear with age in adolescence due to brain maturation.

Voxelwise main effects of group, age and group-by-age interactions were examined by regression of the general linear model (GLM). Significant clusters were identified using threshold-free cluster enhancement (Smith and Nichols, 2009) with the threshold for significance set at *p* < 0.05, Family-Wise Error (FWE) correction for multiple comparisons for both whole-brain analysis and in small volumes (i.e., small volume correction, SVC; Friston, 1997; Worsley et al., 1996). The Harvard–Oxford neuroanatomical Atlas was used to label statistically significant clusters (http://neuro.debian.net/pkgs/fsl-harvard-oxford-cortical-lateralized-atlas.html). Clusters smaller than 20 voxels are not reported.

For regions showing group-by-age interactions, estimates of mean tissue volumes were extracted to visualise the effect.

#### 2.3.2 Small volume correction analysis within ACC

The ACC was defined as an ROI given the consistency with which structural differences are observed in this region in a meta-analysis of studies of adult MDD (Bora et al., 2012). The ACC was anatomically defined using the atlas for automated anatomical labelling (AAL; (Tzourio-Mazoyer et al., 2002)) within MRICro (http://www.mccauslandcenter.sc.edu/mricro/). Within the AAL atlas, templates for “Cingulum_Ant_R” and “Cingulum_Ant_L” were combined to delineate a bilateral ACC ROI.

Voxelwise main effects of group, age, and group-by-age interactions were examined using the same procedures described above. Threshold-free cluster enhancement correction for multiple comparisons was applied within the ACC mask and the level of significance set at *p* < 0.05 FWE corrected.

### 2.4 Post-hoc tests

For regions showing a significant main effect of group or group-by-age interaction, values were extracted to conduct partial correlations with GMV, controlling for age and gender, first across the entire cohort and then within each group separately. These tests were conducted to examine the nature of the relationship between any volumetric differences observed, irrespective of age and gender, either across the whole cohort or within each group alone. Within regions showing a significant relationship with group, age, or group-by-age interaction, values were extracted to conduct within-group linear regressions independently with SMFQ and STAI-T in the MDD group only, controlling for the confounding influence of gender and age. These tests were conducted to ascertain the influence of severity of depression or anxiety on GMV irrespective of patient sex or age.

## 3 Results

### 3.1 Group differences in the ACC

Prior to running analyses, the effect of medication was examined within the depressed group only. No effect of medication was observed in the ACC. Furthermore, medication use was not observed to show an interaction with either gender or age (or age covarying for gender) in the ACC. A decision was therefore made to combine the medicated and unmedicated depressed groups into a single MDD group for all subsequent analyses detailed below.

#### 3.1.1 Main effect of group

No significant main effect of group was observed in the pregenual region of the ACC (Table S1 available online).

#### 3.1.2 Main effect of age

**Fig. 1:**
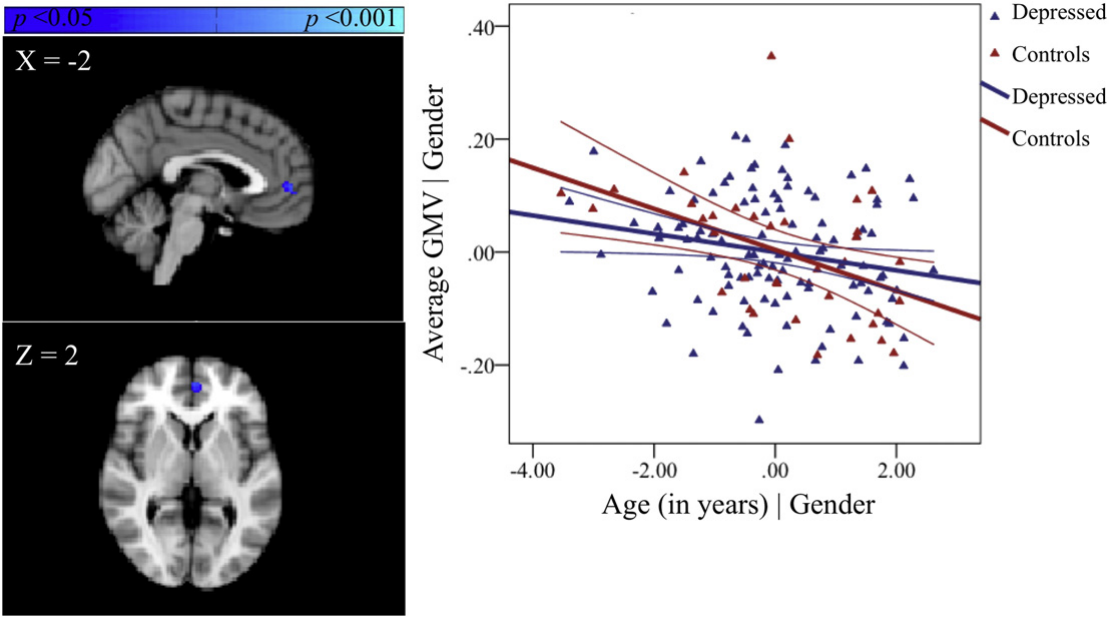
Regions showing age-related decreases in GMV for both groups in the ACC, covarying for gender and associated partial regression plot with 95% confidence intervals. The values shown on *x*- and *y*-axes respectively represent residualised age and residualised average GMV.

There was a significant negative relationship between GMV and age in the left paracingulate gyrus (Fig. 1, Table S1, available online).

#### 3.1.3 Group-by-age interaction

**Fig. 2:**
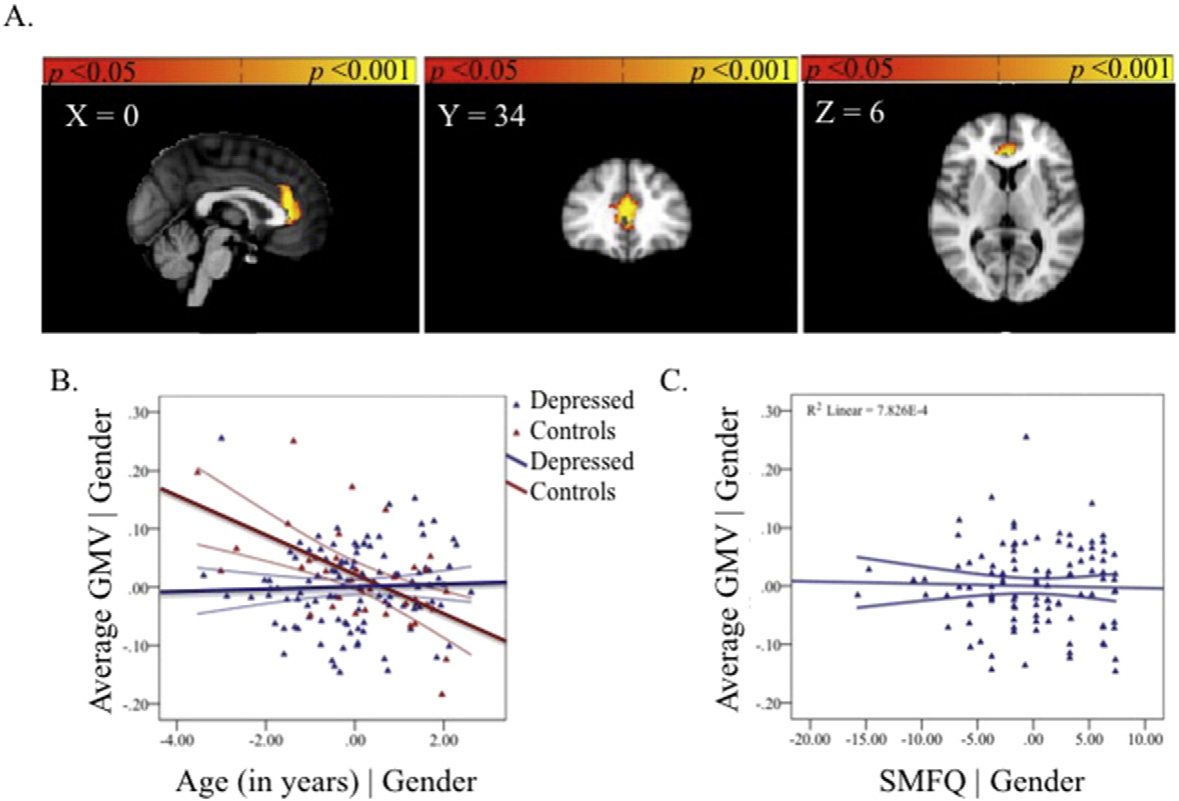
The group-by-age interaction in the ACC (A). Partial regression plot showing the between-group relationship with age, covarying for gender. The values shown on *x*- and *y*-axes respectively represent residualised age and residualised average GMV (B). Post-hoc within-group partial regression with self-reported depression severity, covarying for age and gender. The values shown on *x*- and *y*-axes respectively represent residualised depression scores and residualised average GMV. Adolescents with MDD showed no significant correlation between GMV in the ACC region and severity of depression (*r* = −0.03, *p* = 0.77) (C). Each plot also includes 95% confidence intervals.

A significant group-by-age interaction was observed in ACC and confined to the pregenual region. The region showing a group-by-age interaction did not overlap with the region showing a main effect of age. Depressed adolescents showed no significant change in GMV with age in this region whereas the healthy adolescents showed a reduction (Fig. 2, Table S1, available online).

### 3.2 Group differences across the whole-brain

Prior to running analyses, the effect of medication was examined within the depressed group only. No effect of medication was observed at the level of the whole-brain. Furthermore, medication use was not observed to show an interaction with either gender or age (or age covarying for gender) in any region across the whole-brain. A decision was therefore made to combine the medicated and unmedicated depressed groups into a single MDD group for all subsequent analyses detailed below.

#### 3.2.1 Main effect of group

No significant main effect of group was observed in GMV across the whole-brain (Table S1, available online).

#### 3.2.2 Main effect of age

**Fig. 3:**
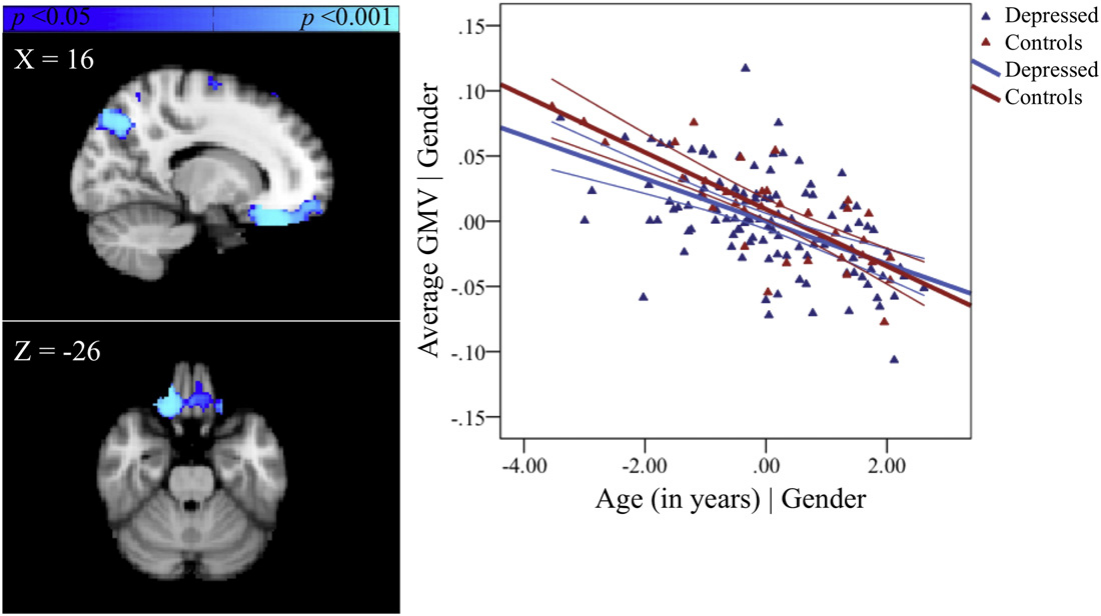
Regions showing age-related decreases in GMV for both groups across the whole brain, covarying for gender and associated partial regression plot with 95% confidence intervals. The values shown on *x*- and *y*-axes respectively represent residualised age and residualised average GMV.

No significant positive relationship with age was observed in GMV across the whole-brain. However, a significant negative relationship with age was observed in several grey matter regions including the right orbital frontal cortex extending to the pgACC (Fig. 3, Table S1, available online). Other regions showing a significant negative relationship with age are reported in Table S1, available online.

#### 3.2.3 Group-by-age interactions

**Fig. 4:**
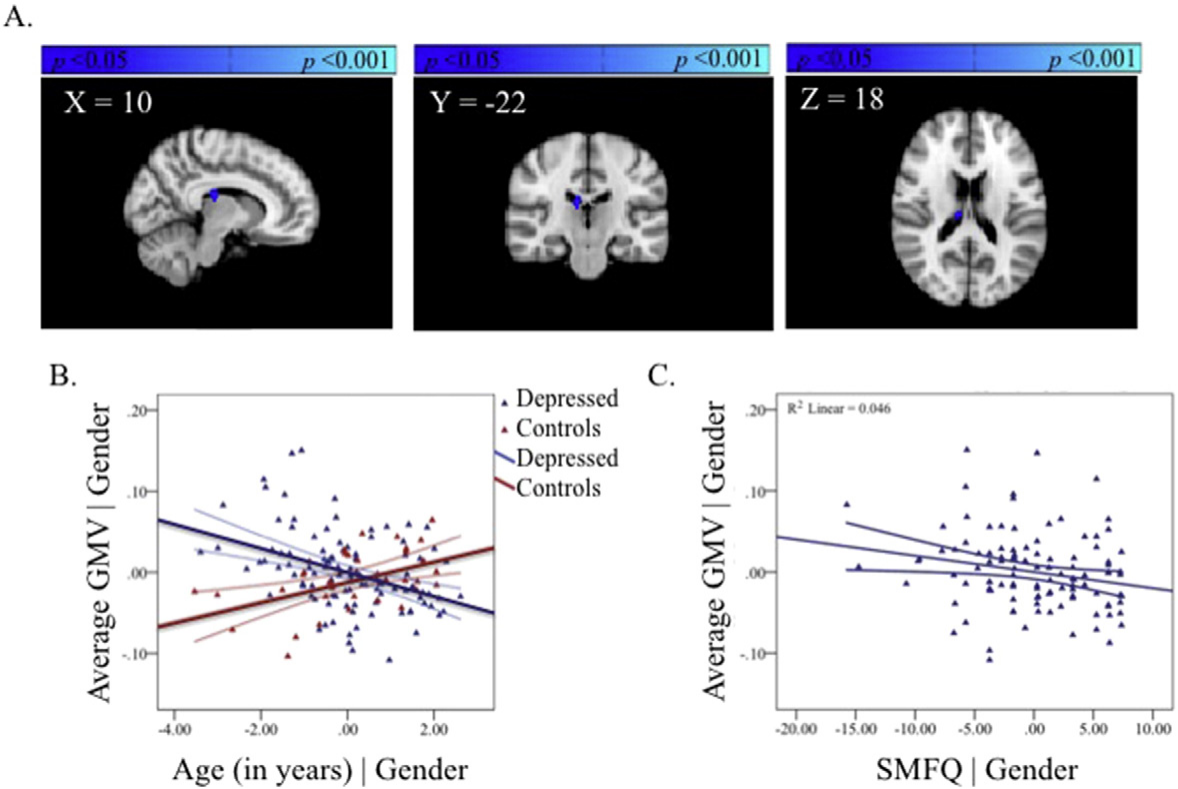
The group-by-age interaction in the thalamus (A). Partial regression plot showing the between-group relationship with age, covarying for gender. The values shown on *x*- and *y*-axes respectively represent residualised age and residualised average GMV (B). Post-hoc within-group partial regression with self-reported depression severity in the depressed group, covarying for age and gender. The values shown on *x*- and *y*-axes respectively represent residualised depression scores and residualised average GMV. Adolescents with MDD showed a significant within-group negative correlation between GMV in the thalamus and self-reported depressive symptoms (SMFQ *r* = −0.21, *p* = 0.03) (C). Each plot also includes 95% confidence intervals.

A significant group-by-age interaction was observed in the thalamus. Depressed adolescents showed a mean reduction in GMV with age in this region whereas the healthy adolescents showed a mean increase with age (Fig. 4, Table S1, available online). The region showing a group-by-age interaction did not overlap with any region showing a main effect of age.

### 3.3 Post-hoc correlations

#### 3.3.1 GMV

Controlling for age and gender, partial correlations with GMV were examined in regions showing a significant group-by-age interaction (i.e., ACC and thalamus), first across the entire cohort and then within each group separately. When controlling for gender and age, GMV in the ACC and thalamus were significantly negatively correlated across the whole cohort (*r* = −0.20, *p* = 0.02), however, were not significantly correlated when looking at either group alone (MDD: *r* = −0.07. *p* = 0.50; Control: *r* = −0.19, *p* = 0.28).

#### 3.3.2 Severity of symptoms

Correlations with symptom severity were examined in regions showing a significant group-by-age interaction (i.e., ACC and thalamus) using partial linear regression in the depressed group only controlling for age and gender.

#### 3.3.3 Anterior cingulate cortex

**Supplementary Figure 1:**
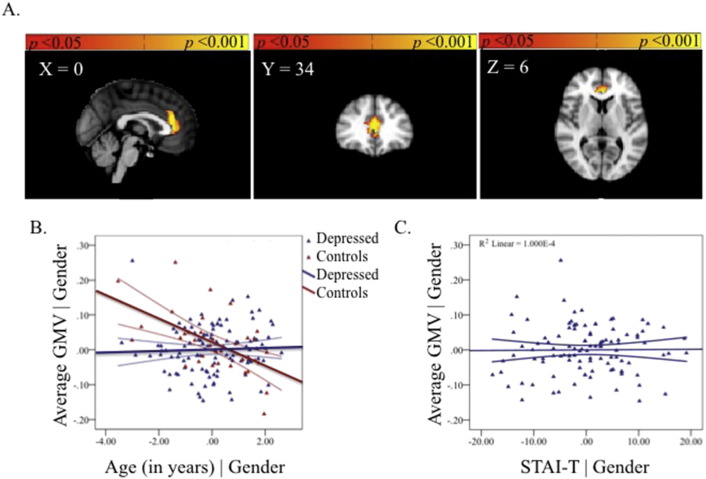
The group-by-age interaction in the ACC (A). Partial regression plot showing the between-group relationship with age, covarying for gender. The values shown on *x*- and *y*-axes respectively represent residualised age and residualised average GMV (B). Post-hoc within-group partial regression with self-reported trait anxiety severity in the depressed group, covarying for age and gender. The values shown on *x*- and *y*-axes respectively represent residualised anxiety scores and residualised average GMV. GMV in the ACC region did not significantly correlate with level of trait anxiety (*r* = 0.01, *p* = 0.92) (C). Each plot also includes 95% confidence intervals.

Adolescents with MDD showed no significant correlation between GMV in the ACC region and severity of depression (*r* = −0.03, *p* = 0.77) (Fig. 2). Furthermore GMV in that region did not significantly correlate with level of trait anxiety (*r* = 0.01, *p* = 0.92) (Fig. S1, available online).

#### 3.3.4 Thalamus

**Supplementary Figure 2:**
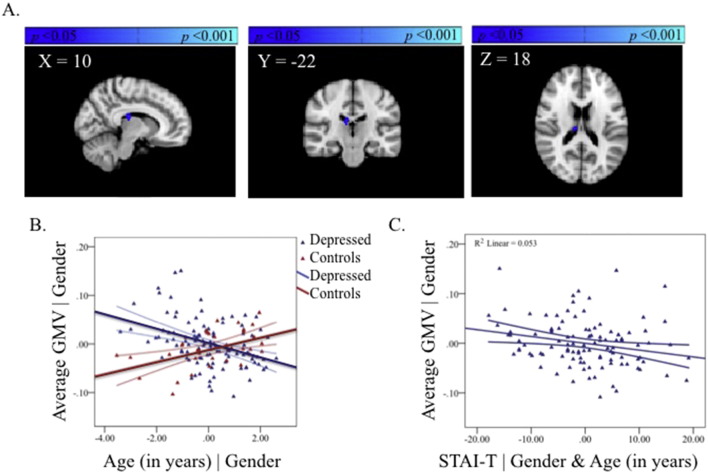
The group-by-age interaction in the thalamus (A). Partial regression plot showing the between-group relationship with age, covarying for gender. The values shown on *x*- and *y*-axes respectively represent residualised age and residualised average GMV (B). Post-hoc within-group partial regression with self-reported trait anxiety severity in the depressed group, covarying for age and gender. The values shown on *x*- and *y*-axes respectively represent residualised anxiety scores and residualised average GMV. A significant negative correlation with level of trait anxiety was also observed (STAI-T *r* = − 0.23, *p* = 0.02) (C). Each plot also includes 95% confidence intervals.

Adolescents with MDD showed a significant within-group negative correlation between GMV in the thalamus and self-reported depressive symptoms (SMFQ *r* = −0.21, *p* = 0.03; Fig. 4). A significant negative correlation with level of trait anxiety was also observed (STAI-T *r* = −0.23, *p* = 0.02) (Fig. S2, available online).

## 4 Discussion

Our hypothesis that MDD patients would show significant reductions in GMV in the ACC compared with controls was not supported. This null result was obtained despite, to our knowledge, this being the largest group of adolescents with current MDD studied to date. The alternate hypothesis that there would be an effect of age on group differences was supported. Thus relative to age-matched healthy controls, younger patients with current depression show a dissimilar pattern of GMV differences across age means compared to older patients with current depression in the ACC (see Fig. 2B). We cautiously interpret these cross-sectional data as suggesting that in depression, age may index a set of latent factors that are operating differentially in the younger more immature and older maturing adolescent brain, although further longitudinal research is required to firmly attest to such a claim. The ACC in depressed adolescents may therefore be age-sensitive and have prognostic effects. The presence of an interaction giving opposite effects by age are very likely accounting for the absence of an overall group difference between adolescents with current depression and healthy controls. Interestingly, post-hoc testing in the MDD group indicated that the group-by-age interaction in pgACC was unrelated to current self-reported symptoms (see Fig. 2C). This suggests that age rather than subjective severity of illness may be a better index of GMV differences in the depressed adolescent brain.

In contrast, and unexpectedly, we showed that adolescent patients with current MDD compared to healthy controls also show an interaction between age-related differences in GMV in the thalamus. While the cross-sectional nature of the data render it difficult to know the true direction of these differences, adolescents with depression show an opposing pattern of GMV changes with age relative to healthy controls (see Fig. 4B). The presence of significant within-group correlations between GMV in the thalamus and self-reported depressive symptoms supports the possibility of an effect of depressive severity on this region in adolescents with current MDD (see Fig. 4C). When controlling for age and gender, GMV in thalamic and ACC regions showing a significant group-by-age interaction did not correlate with one another in the MDD group alone, suggesting that in adolescence these regions may be related to different aspects of the illness. Given the vast clinical heterogeneity of the disorder, larger samples examining subtypes of MDD may prove more fruitful. Indeed, the presence of a significant correlation between thalamic and ACC GMV across the entire cohort of adolescents suggests that larger samples may be needed when examining correlations between regions showing different relationships between GMV and age.

The location of the results in the ACC was similar to those reported in depressed adults (Bora et al., 2012). Here, only the pregenual region of the ACC showed significant GMV interactions between group and age, with controls showing a dissimilar pattern of GMV differences across age compared to depressed patients. While the significance of this dissimilarity is unclear, one potential explanation is that MDD is preferentially occurring in the presence of a more mature ACC, particularly in early adolescence. Other possible explanations are that adolescents with MDD have delayed or abnormal brain development relative to healthy controls and do not show the normative pattern of GMV reduction in the ACC over time. Again however as these results are cross-sectional, longitudinal replication is needed in order to accurately chart the developmental trajectory of the ACC in adolescents with and without MDD. Contrary to the structural imaging literature in adult MDD (Bora et al., 2012) small volume correction of the ACC was necessary to detect differences in case–control relationships with age in this region, suggesting that observable (at the whole-brain level) between-group VBM differences emerge over time after symptoms emerge. This explanation is in line with the failure of Whittle and colleagues (Whittle et al., 2014) to observe significant group-by-time interactions in a community sample of adolescents prior to and following their first depressive episode. GMV in the pgACC was not significantly correlated with either severity of current depression or level of trait anxiety, perhaps attributable to the relative stability of GMV in this region across age in the MDD group.

### 4.1 The thalamus as a locus of adolescent MDD

The significant post-hoc correlation between self-reported severity of depression and anxiety and thalamic reductions in GMV in the MDD group (see Fig. 4C) suggests that, in adolescence, volumetric reductions of thalamic grey matter are associated with the quality of the MDD episode. Volumetric differences in the thalamus are not commonly reported in structural studies of adults with MDD (Bora et al., 2012), yet a recent study on a large cohort of adults with current MDD reported volumetric reductions in bilateral thalamus (Nugent et al., 2013). Postmortem studies show an elevated neuronal density in the mediodorsal nucleus of patients with MDD, but not in healthy subjects or subjects with bipolar disorder or schizophrenia (Young et al., 2004). The thalamus has been suggested to undergo a significant amount of reorganisation from early childhood through adolescence to early adulthood (Lebel et al., 2008). Thus, in adolescents with MDD the change in GMV with age may reflect reorganisation of this region rendering detection of group differences in GMV difficult. Despite the paucity of research showing volumetric differences in the thalamus, there is good evidence for the persistence of functional changes in the thalamus associated with adult MDD (Graham et al., 2013; Hamilton et al., 2012). While the coarse spatial resolution of whole-brain structural neuroimaging precludes localisation of our results to thalamic nuclei or a single nucleus of the thalamus, pulvinar and medio-dorsal nuclei are spatially consistent with the result observed.

### 4.2 The emergence of GMV deficits in the anterior cingulate

In contrast to the unanticipated findings in the thalamus, MDD adolescents showed a pattern of age-related differences in GMV in the pgACC distinct to healthy controls (see Fig. 2B), as expected from large-scale studies of normal brain maturation (Paus, 2005). Other studies of adolescents with depression have failed to find significant group-by-time interactions in ACC (Whittle et al., 2014), but this may be attributable to the comparatively smaller sample size of adolescent patients. Given the vast clinical heterogeneity in MDD, large numbers of patients may be necessary before significant group differences can be observed. The pgACC is a component of both the fronto-limbic system and the medial network within CSPT circuitry (Price and Drevets, 2012; Drevets, 2001; Mayberg, 2003; Seminowicz et al., 2004) and is suggested to link cognitive with emotional processes (Mayberg, 1997). Unlike in adults (Bora et al., 2012), adolescents with MDD do not display group differences in GMV in pgACC relative to healthy adolescents (cf. Whittle et al., 2014), suggesting that the aberrant links between cognitive and emotional processes in depression emerge over time. The relatively stagnant pattern of GMV with age in pgACC (see blue line in Fig. 2B) may also explain the absence of a relationship between GMV and symptoms of either depression or anxiety in this region.

Despite total brain volume (encompassing grey matter, white matter and cerebrospinal fluid) remaining constant across adolescence (Lebel et al., 2008), different patterns of brain development occur in different regions of the brain and these maturational changes coupled with individual differences in neurodevelopment may also hinder the detection of group differences in brain structure between cases and controls during this time period (Andersen et al., 2008). For example, the medio-dorsal nucleus of the thalamus is strongly interconnected with the ACC (Klein et al., 2010) and is associated with an accelerated rate of change in fractional anisotropy relative to the cingulate (Lebel et al., 2008) in adolescence. An implication from the current findings could be that the effects on the thalamus, by adult life, influence interconnecting regions including the pgACC.

## 5 Conclusions and study limitations

In conclusion, MDD patients do not show significant reductions in GMV in the ACC compared with healthy controls, contrary to our primary hypotheses. This null finding is unlikely to be attributable to insufficient power given the large sample of adolescents with current MDD examined in the study. More exploratory whole-brain examinations of the data suggest that the emergence of MDD in adolescence is associated with opposing patterns of age-related differences in the thalamus relative to healthy adolescents (see Fig. 4B). GMV in our a priori regions of interest, the pgACC, also showed dissimilar patterns of age-related differences for adolescents with MDD relative to healthy adolescents (see Fig. 2B). We propose that disruption to these regions is linked (either consequentially or in the exacerbation of prepotent differences) to a cascade of further dysfunction to CSPT and fronto-limbic circuits thereby altering the course of healthy brain development. We also show that, irrespective of gender and age, illness severity is correlated with volume reductions in the thalamus (see Fig. 4C), a relationship not observed in the pgACC in adolescence (see Fig. 2C). One benefit of studying MDD during this developmental window is that it permits examination of the disorder nearer the onset with fewer confounding influences, thereby providing better clues into its aetiology.

Strengths aside, our study is not without limitations. First and foremost, the cross-sectional nature of our data do not permit the examination or comparison of developmental trajectories across groups. Nonetheless, we believe that cross-sectional studies can be both useful and informative, especially with respect to providing the foundation of information where specific hypotheses can be generated and tested in future longitudinal research. Second, onsets of depression have been hypothetically linked to time-sensitive disruption of regionally-specific brain maturational cascades (Andersen and Teicher, 2008). Although approximately 93% of the adolescent patients with depression included in this sample were experiencing their first episode, we lack reliable information about the onset timing of depressive illness in cases where the current episode is not the first episode and thus are unable to examine the effect MDD onset on brain maturational processes as was done in a recent seminal study (Whittle et al., 2014). We also lack information with respect to timing of patient stressors and quality of rearing environment, which limits our ability to test respective hypotheses of a neurodevelopmental cascade and/or psychosocial acceleration (where unstable rearing environments accelerate development; Belsky and Pluess, 2013) underlying depression in adolescence. Age in this cross-sectional cohort may therefore constitute a proxy mechanism for examining the moderating and mediating influences of genetic and epigenetic vulnerability. For example, exposure to early adversity could accelerate onsets of MDD and help to differentiate early- from mid-to-late-adolescence onsets of MDD (Andersen and Teicher, 2008). Under this purview, adolescence would constitute a critical neurodevelopmental period for the emergence of unipolar depressions exposed to suboptimal family environments and result in a differential course for these illnesses through its effects on the developing brain and mind (Kuyken and Dalgleish, 2011; Walsh et al., 2012). Third, approximately one third of our patient sample had been taking medication prior to the scan. Combined with the knowledge that at least five adolescent patients with MDD were experiencing a recurrent form of depression at the time of scanning, and that we lacked first episode versus recurrent data from 14 patients, the possibility remains that the heterogeneity of our sample precluded the observation of group differences in GMV or weakened any results observed. Finally, we chose not to correct for either TIV or total GMV in this developmental sample, given that systematic cross-sectional or longitudinal studies of such correction have not been performed in this age-range (Mills and Tamnes, 2014). Correction for TIV or total GMV may be suboptimal due to nonlinear changes in total GMV across adolescence (Hedman et al., 2012) and region-specific maturational heterochronicity (Paus et al., 2008) leading to a potential bias in results (Barnes et al., 2010). Nonetheless, it is possible that correction for TIV or total GMV (in addition to age and gender) would have altered the observed results. Limitations notwithstanding, future work will include longitudinal behavioural and clinical follow-up to better understand the aetiology and developmental progression of MDD in adolescence and to provide insight into neurobiological predictors of treatment outcome. We hope that a better understanding of the pathophysiology of depression in adolescence will help to identify ways to alter the natural course of illness relapse and recurrence.

The following are the supplementary data related to this article.

Supplementary Table 1 Brain regions showing statistically significant differences in grey matter volume between the depressed group and healthy control group in the ACC ROI and across the whole-brain. Clusters larger than 20 voxels and surviving *p*< 0.05 FWE correction for multiple comparisons are reported.

Supplementary data to this article can be found online at http://dx.doi.org/10.1016/j.nicl.2014.12.019.

## Conflicts of interest

Professor Bullmore is a part-time employee of GlaxoSmithKline. Professor Sahakian consults for Cambridge Cognition, Servier and Lundbeck. She holds a grant from Janssen/J&J. Professors Goodyer and Suckling, Drs. Hagan, Graham, van Nieuwenhuizen, Lennox, Ooi, Simas, Tait, Whitaker, and Mr. Widmer report no competing interests.

## Figures and Tables

**Table 1:** Demographic and clinical characteristics of the depressed and healthy adolescent groups.

	Healthy adolescents *n* = 36	Depressed adolescents *n* = 109	*F* or χ^2^	*p*
Mean Age (SD), *range*	15.65 (1.45), *12.14–17.73*	15.56 (1.27), *11.83–17.96*	*F* = 0.831	*p* = 0.722
% female	72.22%	74.31%	χ^2^ = 0.061	*p* = 0.805
% right-handed	91.67%	87.16%	χ^2^ = 0.532	*p* = 0.466
IQ (available from 22 patients and 36 controls)	100.94 (10.93)	96.59 (11.45)	*F* = 0.615	*p* = 0.436
Mean SMFQ score	2.97 (1.92)	17.46 (5.07)	*F* = 28.501	*p* < 0.001
Mean STAI state score	29.53 (6.45)	45.64 (10.90)	*F* = 10.633	*p* < 0.001
Mean STAI trait score	31.03 (6.39)	60.37 (8.08)	*F* = 3.225	*p* < 0.001
% first MDD episode (available from 90 patients)	–	93.33%		
Antidepressant medication use:				
Fluoxetine	–	29.36%		
Citalopram	–	3.67%		
Propanolol	–	0.92%		
Sertraline	–	0.92%		
Other medications used:				
Risperidone	0	2.75%		
Cetirizine	0	1.83%		
Doxycycline	0	1.83%		
Gabapentin	0	0.92%		
Mefenamic acid	0	0.92%		
Tetralysal	0	0.92%		
Pizotifen	0	0.92%		
Levothyroxine	0	0.92%		
Prednisone	0	0.92%		
Ranitidine	0	0.92%		
Lamotrigine	0	0.92%		
Zolpidem	0	0.92%		
Circadin	0	0.92%		
Diazepam	0	0.92%		
Mebeverine	0	0.92%		
Fenagon	0	0.92%		
Infliximab	0	0.92%		
Mesren	0	0.92%		
Azathioprine	0	0.92%		
Asthma medication	0	1.83%		
Pentasa	2.78%	0		
Ibuprofen	2.78%	0		
