# Supplementary material for: Adolescents with current major depressive disorder show dissimilar patterns of age-related differences in ACC and thalamus
Source: Neuroimage Clin. 2015 Jan 7;7:391–9. doi: 10.1016/j.nicl.2014.12.019 (PMC4309951; doi:10.1016/j.nicl.2014.12.019)
Supplement: Supplementary Table 1 — Brain regions showing statistically significant differences in grey matter volume between the depressed group and healthy control group in the ACC ROI and across the whole-brain. Clusters larger than 20 voxels and surviving p< 0.05 FWE correction for multiple comparisons are reported. [file mmc1.pdf]

Table S1. Brain regions showing statistically significant differences in grey matter volume between the depressed group and healthy control group in the ACC ROI and across the whole-brain. Clusters larger than 20 voxels and surviving p<0.05 FWE correction for multiple comparisons are reported. Coordinates represent the maximum intensity voxel of the cluster in MNI standard space. The brain locations of coordinates are designated using the Harvard-Oxford Cortical and Subcortical Structural Atlases.

Grey Matter Comparison	# of voxels	*p*-value	MNI Coordinates	Brain region
**ACC ROI**				
Depressed>Control	-	n.s.	-	-
Control>Depressed	-	n.s.	-	-
Age Depressed>Age Control	583	<0.004	0, 34, 6	Anterior cingulate gyrus
Age Control>Age Depressed	-	n.s.	-	-
Positive relationship with Age	-	n.s.	-	-
Negative relationship with Age	35	<0.003	-2, 52, 2	Left paracingulate gyrus
**Whole-brain**				
Depressed>Control	-	n.s.	-	-
Control>Depressed	-	n.s.	-	-
Age Depressed>Age Control	-	n.s.	-	-
Age Control>Age Depressed	28	0.037	10, -22, 20	Right thalamus
Positive relationship with Age	-	n.s.	-	-
Negative relationship with Age	4677	0.001	16, 28, -26	Right orbital frontal cortex
	3223	0.001	10, -76, 40	Right precuneus
	2993	0.005	-10, 30, 60	Left superior frontal gyrus
	2173	0.002	-52, -28, 42	Left supramarginal gyrus
	715	0.008	42, 32, 20	Right middle frontal gyrus
MNI = Montreal Neurological Institute				
n.s. = no significant results				
